# The Teutonic Health Resorts: British Substitutes That Need Awakening

**Published:** 1915-05-29

**Authors:** 


### "THE TEUTONIC HEALTH RESORTS."

### British Substitutes that Need Awakening.

A paper recently contributed by Dr. Leonard Williams to the Balneological and Climatological Section of the Royal Society of Medicine has been published as an independent pamphlet. Manifestly it is an attempt to meet a position created by the war. Whatever be the actions of other peoples, it is impossible to believe that British subjects will for many years wish to visit either German or Austrian health resorts; nor, for the matter of that, are they likely to be welcome there. Hence, if the welfare of certain types of individuals is to be secured, and the convenience of some who are not invalids consulted, it is necessary to find alternatives which do not appal British patriotism. The enterprise, then, is a laudable one; and even those who may find themselves unable to follow Dr. Williams in all his recommendations, owe him some recognition for the task he has undertaken. He has certainly put into a brief form a large amount of information useful and even necessary to those who have to give advice to patients on the subject of health resorts, and he has presented this with much literary grace and vivacity. Sad to say, his general conclusion is that they do these things better in France. He allows all the virtues of the English spa physician, but maintains that completeness of change, a more gracious and more amusing society, a decided novelty in therapeutic methods, and a position more remote from domestic and business cares, combine to outweigh the claims of the home stations. In support of his opinion he quotes the chalybeate virtues of Tunbridge Wells, now altogether derelict, Cheltenham in a state of animated suspense, and Leamington in a condition of suspended animation. Only patriotism restricts him from extending a specific indictment to other individual localities, while regretfully he includes all under a general condemnation as lacking in something—"enterprise," or what you will. With the exception of Bath as a winter thermal station the British resorts, he concludes, have nothing to offer which France does not supply, "in quantity much greater and in quality much superior." To those therefore to whom a choice is open—and these, indeed, are the persons for whom new resorts have to be found—there is necessarily a special interest in Dr. Williams's pamphlet. For various reasons they have been in the habit of going abroad, and many of them will wish to continue the custom. Here, then, is help for persons so situated. Yet in the existing circumstances it may be doubted whether there will be any widespread desire on the part of invalids to visit the Continent, and therefore we are glad to note that Dr. Williams examines also the virtues of our home stations. He will not give them the first place, but he is free to allow their virtues. And as, whatever he says, many people will stay at home, and some must, it is something to know that we are not wholly destitute. Indeed, when we read what we are told on this subject, and learn at the same time that the French are better, we realise how very good the French must be.

What can be said for Harrogate? Its physicians have distinguished themselves by their earnest, interesting, and illuminating work; there is scarcely any form of treatment—balneary, electrical, or mechanical—which cannot be obtained there; the results in the gouty and submetabolised type of individual are eminently satisfactory; the various springs offer a very wide choice of therapeutic worth; the old well has very many virtues as a stimulant of the liver—in short, no better substitutes for a Teutonic gouty spa can be found than this pleasant station on the Yorkshire moors.

Next on the list is Buxton. Here, also, the physicians have added materially to the stock of general knowledge on matters balneological; the bathing installation is very complete; the climate bracing, with efficient shelter on the north and east. For patients suffering from defective elimination, as opposed to deficient metabolism, Buxton is the spa *par excellence*, and patients victimised by fibrositis, myalgia, or rheumatoid arthritis, or who require their kidneys flushed or their blood cleansed, will find that their appeals to the *genius loci* are not in vain.

A third station competent to deal with such vices of general metabolism as gout and rheumatism is Llandrindod Wells, where the equipment and installation of the baths are excellent, and modern methods are well and carefully applied. The climate is particularly agreeable both to those in health and to the debilitated and neurasthenic type of invalid, while the treatment is well adapted to the atonic, gouty, and rheumatic.

Dr. Williams has some vigorous things to say of the Nauheim cult, though he allows that effervescing baths have some value in certain cardiac disorders. These baths can be obtained at many places in France, and, indeed, in this country also; and Sidmouth is mentioned as a station particularly suitable by its climatic virtues both to the cardiac invalid and to the convalescent and debilitated. Here the baths are prepared by diluting the sea water, and adding carbonic acid. Llangammarch Wells, in mid-Wales, is, we imagine, a less familiar name, but Dr. Williams tells us that as a cardiac spa it possesses possibilities, and that its barium waters are of service in many vascular conditions.

Of the stations with strong brine waters Droitwich and Woodhall Spa are well worthy of attention. They have various climatic and local virtues, and, while mainly utilised in myalgic, neuralgic, and arthritic disorders, they have value also in certain gynæcological troubles. Woodhall Spa, indeed, in virtue of the bromo-iodine content of its waters, is pronounced to be an admirable substitute for the well-known Kreuznach.

From this brief sketch, our British stations, it will be seen, come well out of the trial, even though they have serious rivals in France. It may induce our readers to consult Dr. Williams's pamphlet, and we are sure they will not regret the experience.

